# Thermal Softening Measurements of Refractory High-Entropy Alloys

**DOI:** 10.3390/ma17235718

**Published:** 2024-11-22

**Authors:** Ottó K. Temesi, Albert Karacs, Nguyen Q. Chinh, Lajos K. Varga

**Affiliations:** 1 H-ION Kft., Konkoly-Thege Miklós út 29.-33., H-1121 Budapest, Hungary; otto.temesi@h-ion.hu; 2 Department of Materials Physics, Eötvös Loránd University, Pázmány Péter Sétány 1/A, H-1117 Budapest, Hungary; nguyen.quang.chinh@ttk.elte.hu; 3 Mikrot Kft., Konkoly-Thege Miklós út 29.-33., H-1121 Budapest, Hungary; 4 HUN-REN Wigner Research Centre for Physics, H-1525 Budapest, Hungary

**Keywords:** temperature dependence of mechanical properties, elastic moduli, yield stress and hardness, thermal expansion, indentation depth, softening and allotropic phase transformations, refractory high-entropy alloys

## Abstract

Home-built equipment will be presented able to measure the thermal expansion (with a flat indenter) and indentation depth (with a pointed indenter) up to 1100 °C. In dilatometer mode, the allotropic phase transformations can be studied. For hardness, a Rockwell-type measurement is adopted. First, we apply a small load and measure the displacement consisting of a dominant positive thermal expansion and a small negative indentation depth contribution. Then, we repeat the thermal cycle with such a high load that the compensation appears at around 250–300 °C. With increasing temperature, the indentation depth starts to dominate and we can notice a contraction. The indentation depth as a function of temperature, ID(T), will be obtained by subtracting the high load curve from the low load curve. A new rational fraction expression will be tested to describe the thermal softening of pure metals and refractory HEAs. Still, we are working on improving the equipment to extend the working temperature up to 1200 °C.

## 1. Introduction

In practice, the mechanical resistance against high-temperature softening should be measured and characterized through the temperature dependence of mechanical parameters like elastic moduli, yield stress and hardness. It is difficult and costly to create an experimental environment and measuring technique around and above 1000 degree Celsius. Phenomenological models of mechanical characteristics can lead to financial and time savings. Therefore, a short review of the theoretical models and formulas will be presented, concerning the temperature dependence of elastic moduli, yield stress and hardness.

A good compendium about the elastic modulus estimation models was presented by Weiquo Li et al. [1]. Their formulas follow the general adopted form, and the values at different temperatures are given with the reference of the parameter value at zero absolute temperature:

(1) $$\frac{E(T)}{E(T_{o})}=\frac{{\left(1+\int_{0}^{T_{o}}\alpha (T)dT\right)}^{3}}{{\left(1+\int_{0}^{T}\alpha (T)dT\right)}^{3}}\times {\left(1-\frac{T-T_{o}}{T_{m}-T_{o}+\frac{\Delta_{fus}H}{C_{v}}}\right)}^{1/2}$$

where α is the linear thermal expansion coefficient, *∆_fus_H* is the heat of fusion and *C_v_* is the specific heat capacity. This rational fraction expression was furthermore simplified by Zakarian et al. [2], who deduced the following “universal” temperature dependence for the elastic (Young) modulus:

(2) $$E(T)/E_{o}=1-0.2\frac{T}{T_{m}}-0.25{\left(\frac{T}{T_{m}}\right)}^{2}$$

where *E_o_* is the elastic modulus at zero temperature, *T_m_* is the melting point of the material.

We have checked the “universality” of Equation (2), using the experimental *E* versus *T* data collections of [3] and we have found no matching with the calculated data. Even more, instead of the theoretically predicted concave curve, the experimental points follow a convex curve.

Nevertheless, the “universal” formula correctly predicts the value of *E* at the melting point. Inserting *T = T_m_* into Equation (2), we obtain the correct value of *E* at *T_m_* which is 55% of the initial value, not zero as was supposed by Born and Huang [4] based on theoretical considerations.

Concerning the temperature dependence of yield stress, we cite here Z. Wu et al. [5], who revived the idea of Dietze [6] about the temperature dependence of yield stress, which is the same as that of the Peierls stress [7], and obtained that the yield stress exponentially decaying with temperature:

(3) $$\sigma_{y}(T)\approx \sigma_{y}(0)\cdot \exp\left(\frac{-2\pi \omega_{o}}{bT_{m}}T\right)$$

where *ω_o_* is the dislocation width, which depends linearly on the absolute temperature, with the proportionality factor being 1/*T_m_* and *b* being the Burgers vector.

Others have taken another route [5], assuming that yielding at temperature *T* occurs when the sum of the elastic deformation energy per unit volume and the corresponding heat energy reaches a certain value. The ratio of the yield stresses at temperature *T* compared to its initial value can be calculated without free parameters using the tabulated values of specific heat capacities and of elastic moduli:

(4) $$\sigma_{y}(T)=\sigma_{y}(T_{o})\cdot {\left[\frac{1+\nu (T_{o})}{1+\nu (T)}\frac{E(T)}{E(T_{o})}\left(1-\frac{\int_{T_{o}}^{T}C_{p}(T)dT}{\int_{T_{o}}^{T_{m}}C_{p}(T)dT}\right)\right]}^{1/2}$$

Although both temperature dependencies are related to the hardness, they are not the same. The main difference is that at a melting temperature of about 0.55, part of the modulus is still present, whereas the yield stress (together with the hardness) decays down to zero.

Even more, the yield stress (*YS*) scales with the Vickers hardness (*HV*) at around room temperature are related by the well known three-time relations [8,9]:

(5) HV=3⋅YS

In general, the variation in hardness (*HV*) with *T* is very characteristic: at small temperatures, *HV* is practically constant and as the temperature of the material increases, hardness decreases and at some point, a drastic change in hardness occurs. The hardness at this point is termed the hot or red hardness of that material. Such changes can be seen in materials such as heat-treated alloys. Precipitation hardening may overlap with red hardness behavior; this is why special attention is needed to separate them.

The formula of the variation in hardness with temperature was published by Ito [10,11,12]:

(6) $$\frac{H(T)}{H(T_{o})}=\exp(-BT)$$

where *H*(*T_o_*) is the intrinsic hardness, and B is the softening coefficient of hardness. These two parameters change after the red hardness point. Considering the three-time relation (5), we can accept that the explanation given for the temperature dependence of yield stress is valid for Equation (6) of hardness as well.

The first reliable high-temperature Vickers hardness data (up to 800 °C) were published in 1950 by Westbrook [13]. He proposed another model to describe the hardness versus temperature, The model equation is shown as follows:

(7) $$H(T)=A^{\prime }\exp(-B^{\prime }/T)$$

where $A^{\prime }$ and $B^{\prime }$ are constants, which also have one set of values at a low temperature and another set at a high temperature. The Westbrook model can be derived based on the Arrhenius equation [14,15]. Nevertheless, neither of the two models can be extrapolated to predict the hardness values at lower and higher temperatures. It seems evident that Equations (6) and (7) cannot be valid at the same time for the same sample. In this work, we try to clear up the validity of T dependence relationships and eventually propose a new phenomenological formula.

All the single-phase alloys and compounds showed a common feature of temperature dependence of hardness: a slight decrease to two-thirds of the melting point followed by a drastic decrease indicating that the hardness mechanism had changed. We will refer to the drastic decrease in hardness as “softening” in this work. The first scope of this work is to determine this softening temperature using a home-built thermal displacement meter applying a Rockwell-type hardness data evaluation of indentation depth. Actually, not only is the absolute value of hardness important but so is the relative decrease in it compared to the low temperature value. Actually, we do not claim that we determine the standard compatible value of hardness. The instrument depicts the relative variation of hardness which indicate those characteristic temperatures where some kind of change in the monotonous decrease in hardness happens, like allotropic transformation, or precipitation hardening.

The second scope of this work is to present a formula that is valid in the whole temperature interval and is especially suitable to provide the softening temperature, *Ts*, where the drastic change in *HV* happens.

## 2. Experimental

The traditional hardness measurement is performed at fixed temperatures following a strict time dependence protocol of indentation depth measurements. High-temperature hardness measurements are not often published because of the experimental difficulties. Searching the device market, one can find, for example, the Rtec high-temperature hardness testing equipment of ST Instruments (LE Groot-Ammers, The Netherlands). The instrument provides indents in multiple locations up to 1200 °C and the relatively large chamber permit mounts several samples at the same time (https://www.stinstruments.com, accessed on 1 November 2024).

Nevertheless, the advantage of obtaining similar standard-like hardness data to those at room temperature is overshadowed by the slowness of the measurement, during which structural changes can take place at the high temperature of the measurement. To overcome this problem, in this work, we propose a high heating rate home-built device, with a maximal heating rate of 35 K/min. Actually, we propose a method which measures the indentation depth continuously as a function of temperature. The temperature-displacement data pairs are detected at every second during the heating-up process. The data are stored online and, at the same time, are presented on the screen.

**Figure 1:**
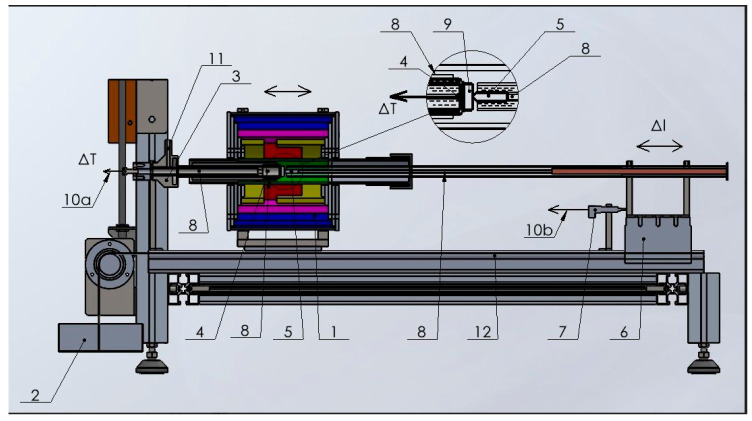
Schematic drawing of the softening measurement. 1. The tube furnace that can be shifted; 2. the stressing load; 3. the fix point for indentation measurement; 4. the thermocouple; 5. the indenter; 6. the guide, moving united gliding on ball bearings; 7. the inductive displacement sensor; 8. rods and tubes made from quartz; 9. the sample; 10. (a) the sample temperature (ΔT), (b) the displacement of the indenter (Δl); 11. the Argon gas inlet; 12. the brittle rail structure.

The schematic drawing of the instrument is shown in Figure 1.

Quartz is the material around the specimen, the sample capture and the indenter guide. See the inserted drawing on the sample and indenter (positions: 4, 5, 8, 9)

**Figure 2:**
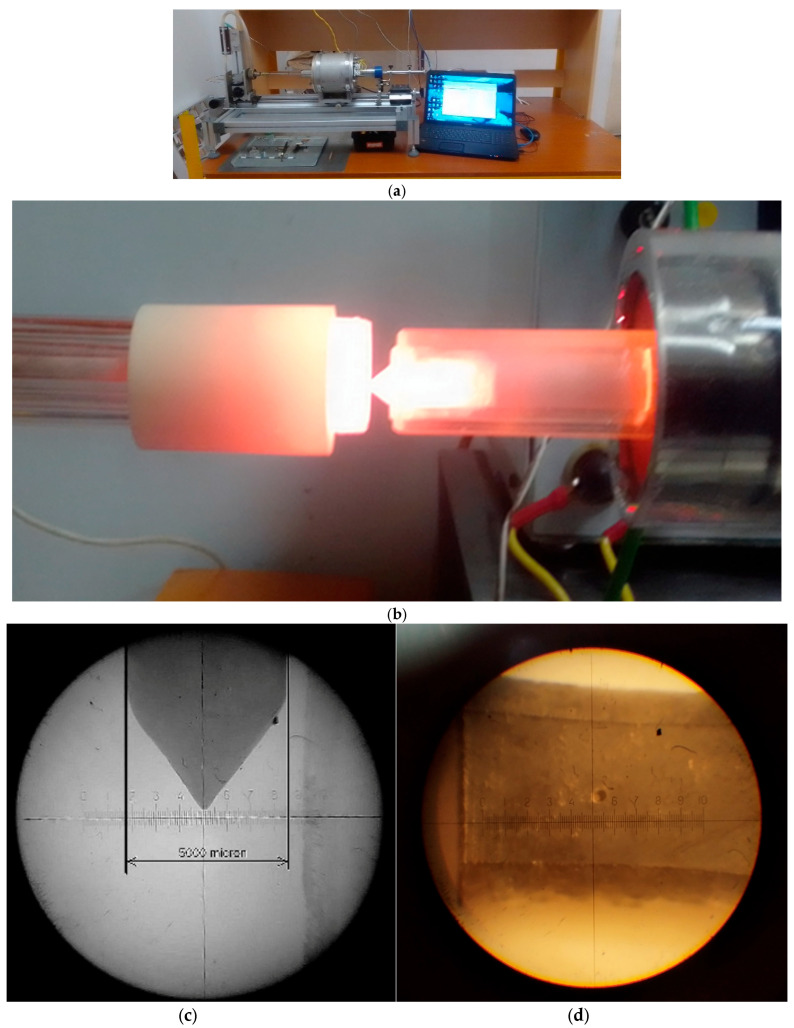
Operational form of the equipment. (**a**) Overall view of the equipment; (**b**) white—hot sample and indenter at around 1000 °C without Ar protection (furnace is shifted); (**c**) the W indenter tip preserving its form after several applications (1 div = 75 µm); (**d**) indent views after indentation with the 2.5 kg load.

The sample, which can be a cube or a cylinder, should not be a regular form, but it is essential to have two plan-parallel faces at a distance of about 5–20 mm for fixed clamping. The section of the faces should be a minimum 5 mm^2^ when we use a 1 mm diameter indenter. The sample (9), the indenter (5) and the clamping quartz tubes (8) form one corpus under a 0.5 kg stressing load. The measuring load is, in general, 1.5–3 kg. The resistive tubular furnace concentrates the heat around the sample, plus or minus 20 mm. Its small size makes possible a high heating rate of up to 35 K/min. The measurement is carried out under flowing argon. Home-made tungsten indenters have been prepared on demand of the particular material and sample size (see Figure 2c).

The trace of the indenter is shown in Figure 2d after 1.5 kg and 0.5 kg loads.

## 3. Results and Discussion

### 3.1. Measurement of Thermal Expansion and Related Thermal Transitions

**Figure 3:**
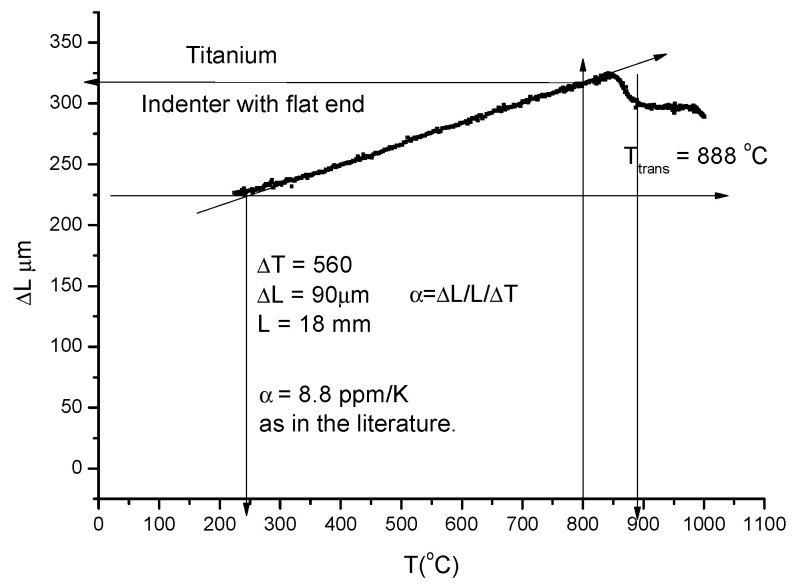
Linear thermal expansion of Titanium and phase transition at T_tr_ = 888 °C.

For thermal expansion measurements, a minimal load (0.5 kg) is applied and an indenter with a flat end was prepared. During this type of measurement, no softening (i.e., no contraction) takes place. More or less, a linear displacement is measured. It should not be forgotten that the overall expansion coefficient includes, beside the expansion of the sample, the expansion of the indenter and the quartz tube as well. These latter expansions can be removed by careful calibration. Nevertheless, the HCP—BCC phase transition of the Titanium sample is clearly visible and can be used for temperature calibration (see Figure 3).

**Figure 4:**
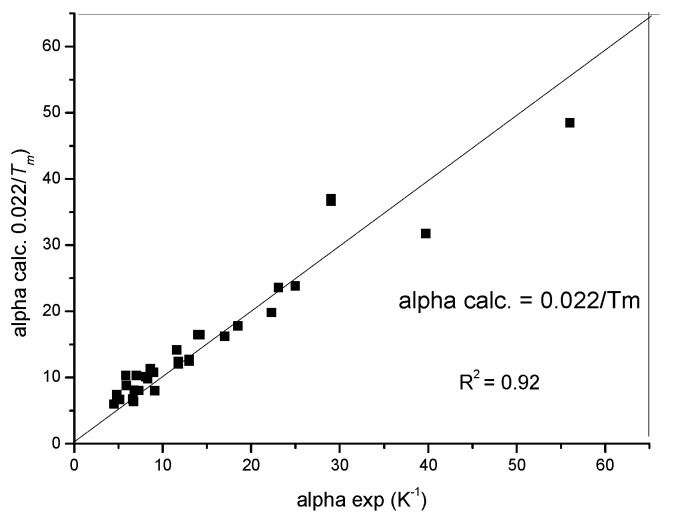
The linear expansion coefficient scales with the reciprocal of the melting temperature.

Driven by curiosity concerning the performance of our home-built device, we measured the thermal expansion of some metals and found a good agreement with the data in the literature; furthermore, we not only detected the phase transformations for the metals showing allotropic phase transformations (for example, for Ti, Zr, Fe and Co), but we also found good agreement between the measured and theoretical transformation temperatures, helping temperature calibration. We found an interesting correlation between the linear thermal expansion coefficient and the melting point: the average linear expansion coefficient (α) above room temperature is inversely proportional to the melting point (*T_m_*) (see Figure 4):

α = (0.022 ± 0.003)/*T_m_* (8)

The relation (8) was verified with R^2^ = 0.92 for 32 elemental metals based partly on our own and partly on data in the literature. The inverse proportionality comes from the expectation that the thermal expansion should be inversely proportional to the atomic bond strength, which is proportional to the melting point. Further discussion of this relationship needs a separate article.

### 3.2. Measurement of Rockwell-Type Hardness as a Softening Meter

**Figure 5:**
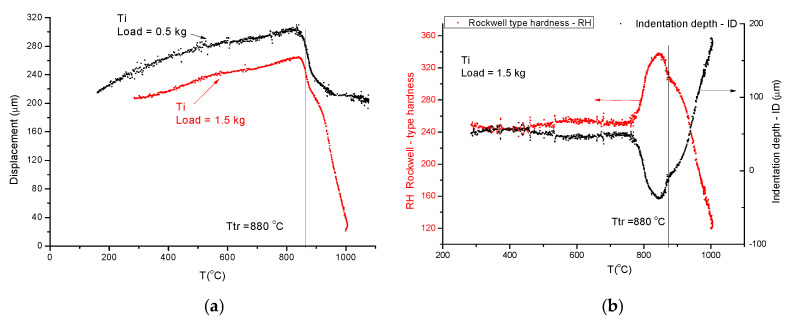
(**a**) Titanium under 0.5 kg and 1.5 kg loads. The red hardness point (*T_m_*/2) was found to be around the phase transition temperature. (**b**) The determination of Rockwell-type hardness (RH) is calculated as RH = 300 − ID using Equation (9).

This time, we use the pointed end tungsten indenter, and we perform two temperature scans: first with a small load to obtain a reference curve, and secondly with a larger load to obtain the measuring curve. The starting temperatures and the heating rate should be the same for the preload of 0.5 kg and for the load of 1.5 kg. Scanning with the larger load, the positive displacement measured by the sensor turns on a negative, indicating the drastic decrease in hardness at high temperatures. The details can be followed on Figure 5. First, we measure the displacement curves for a small load (0.5 kg, in this case) and a larger load (1.5 kg) (see Figure 5a). The difference in these two curves will give the indentation depth (ID) and the Rockwell-type hardness will be obtained (Figure 5b) by subtracting the indentation depth from an arbitrarily chosen number. This time, we have chosen 300:

RH = 300 − ID(9)

After this short presentation of the home-built instrument, we can observe the following advantages compared to the commercially available one: (1) Its high heating rate (short duration of the measurement) makes it possible to avoid the precipitation of phases from the supersaturated solid solution structure of the refractory high-entropy alloys. (2) Irregularly shaped samples with only two plan parallel faces can be measured; there is no need for standard form samples as in the case of the commercial device. (3) Beside the hardness type measurements, it is possible to measure the thermal expansion together with its thermal dependence and, in addition, the phase transition can be studied as it appears in thermal dilatation. This means that our device works as a dilatometer as well. (4) The quick measurement enables series measurements which might be important in a production unit. (5) Last but not least, our home-built device is an order of magnitude cheaper than the commercial device.

**Figure 6:**
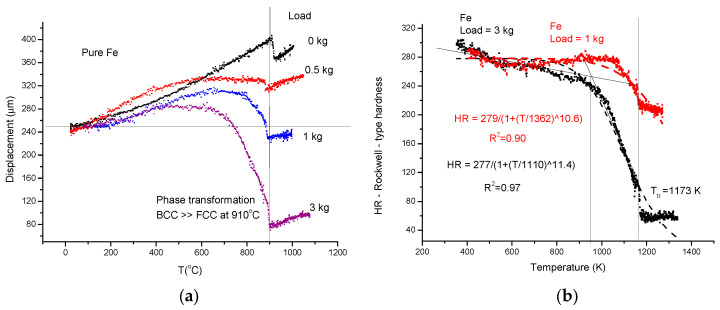
Softening measurement with a high heating rate (30 K/min) on pure iron. (**a**) Temperature dependence of displacement for different loads. (**b**) Rockwell-type hardness as a function of temperature.

In Figure 6, we present the extraction of Rockwell-type hardness from the softening measurements under a pre-load of 0.5 kg and a load of 1.5 kg.

**Figure 7:**
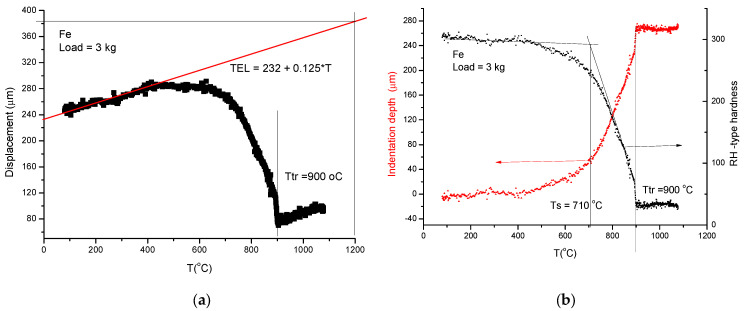
Rapid evaluation protocol for softening measurement on Fe. (**a**) Construction of a thermal expansion line (TEL) based on the initial part of the measured displacement curve (D). (**b**) Representing the indentation depth obtained as ID = TEL − D and the corresponding Rockwell-type hardness RH = 300 − ID.

The measured displacement is a sum of the positive dilatation and negative indentation depth. The hardness (HR) is proportional to the indentation depth (ID). We present two methods for determining ID:

(1) First, applying the so called “traditional” method, we measure the displacement curves (D) for different loads (see Figure 6a). In the spirit of Rockwell-type hardness measurement, we determine the indentation depth (ID) by subtracting two displacement data: those obtained with the larger load (1 and 3 kg) from those obtained with the small one (0.5 kg). The increasing ID is transformed in decreasing HR by simple subtraction of ID from an arbitrarily chosen value (300) (see Equation (9)):
  - The softening temperature (*Ts* = 950 K) is assessed to the “knee” point of the HR(T) curve (see Figure 6b). This hot–red point is visible, displacing to smaller values with increasing load. The optimal load should be chosen as a function of the initial, low-temperature hardness, taking into account that the larger the load, the more difficult it is to perform the measurement.

(2) For rapid measurements, we propose the following protocol:
  - Taking into account that the thermal expansion coefficient, TEC, has a small and negligible increase with temperature, we take as an average TEC the low temperature value and construct in Figure 7, the thermal expansion line (see Figure 7a). The indentation depth (ID) is obtained by subtracting the measured displacement (D) curve from the thermal the expansion line (TEL):

TEL − D = ID(10)

Finally, we obtain the hardness in Figure 7b with Equation (9).

We end this section by presenting the softening measurement on refractory high-entropy alloys presented in Table 1. The samples were prepared in our lab by inductive melting in a water-cooled copper mold. Beside the compositions, some important material characteristics are collected in Table 1 to help interpret the measured softening temperature (*Ts*) data.

**Figure 8:**
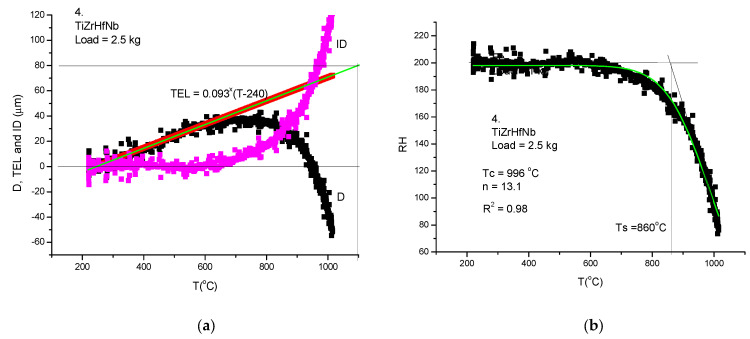
Thermal softening measurement of TiZrHfNb refractory HEA. (**a**) presents the original displacement measurement (D), the thermal expansion line (TEL) determined from the extrapolation of the initial part of the D versus T curve, and the indentation depth (ID) obtained by subtraction ID = TEL − D. (**b**) Rockwell-type hardness is determined by the subtraction RH = 200 − ID and the softening *Ts* temperature is determined only visually from the intersection of two tangent lines.

**Figure 9:**
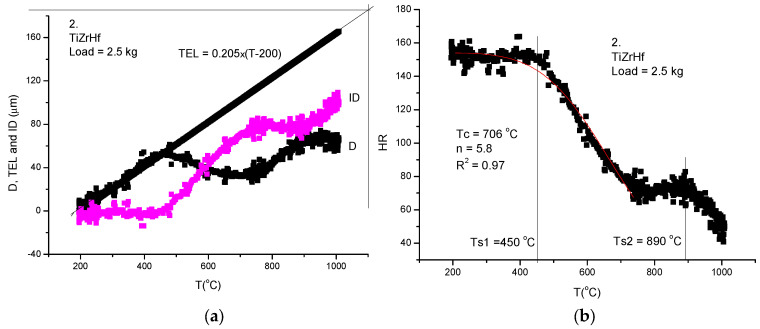
Thermal softening measurement of TiZrHf refractory HEA. (**a**) The parameter ID is calculated following the protocol described above. (**b**) The hardness calculated from 150-ID shows beyond doubt the phase transformation around 800–900 °C, where the second softening starts. The first softening happens at a surprisingly low temperature, *Ts*1 = 450 °C.

To obtain the Rockwell-type hardness, we have applied the rapid measurement technique presented above. We are not going to again present all the steps of the measuring protocol, except for two samples. The sample number 4 is characteristic, in general, for refractory HEAs (Figure 8) and sample number 2 (TiZrHf) shows a double stage decrease as a function of temperature (Figure 9).

The double stage temperature dependence of hardness for our sample number *2*, consisting from three metals (TiZrHf) with allotrope transformations around 800–900 °C, is better revealed after presenting the RH(T) curve (see Figure 9b) calculated from the measured D(T) curve (see Figure 9a).

**Figure 10:**
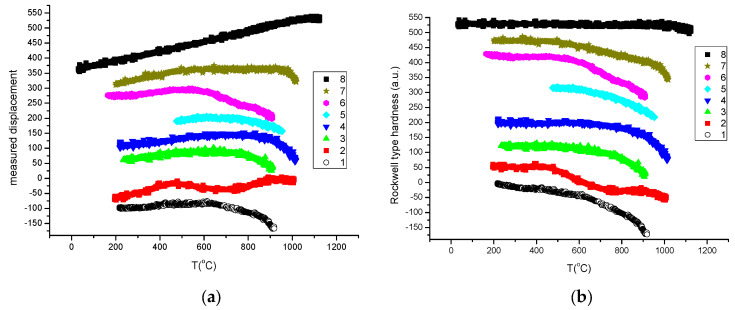
Thermal softening measurements of our refractory HEA samples (see data in Table 1). (**a**) Measured displacement data. (**b**) Calculated RH data.

In Figure 10, we present the measured displacement data (see Figure 10a) and the calculated Rockwell-type hardness for our refractory HEA samples.

In general, the visually determined softening temperature is about half of the melting temperature:

(11) $$T_{s}=\frac{T_{m}}{2}$$

**Figure 11:**
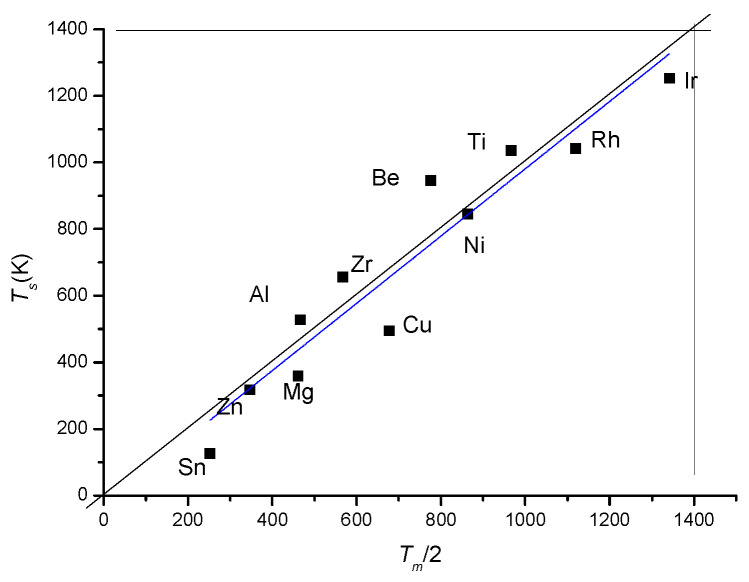
The thermal softening temperature for pure metals is equal to half of the melting point.The fitted (blue) line almost coincides with the first angle bisector (black line).

This relationship is verified for the pure metals, except for those presenting allotrope transformations (see Figure 11). In the case of metals presenting an allotrope transition, the thermal softening temperature decreases to half of the transition temperature, which is much lower than half of the melting point. It turns out that for a true refractory alloy with a high softening temperature, we have to design a structurally stable alloy composition.

Unfortunately, the equations describing the temperature dependence of hardness have a pure goodness-of-fit (R2) measure in the cases of RHEAs. Looking for a better phenomenological equation, we came across the following expression:

(12) $$\frac{H(T)}{H(T_{o})}=\frac{1}{1+{\left(\frac{T}{T_{c}}\right)}^{n}}$$

where $H(T_{c})$ is the reference hardness at room temperature, *T_c_* is the characteristic temperature and *n* is the exponent to be fitted. The *T_c_* and *n* pair data were determined for all eight of our samples and are presented in Table 1. The goodness of fit, R^2^, was always above 0.9.

The characteristic temperature *T_c_* is the temperature where the hardness drops off to be half of its room temperature value (for T = *T_c_*, Equation (12) gives a ratio of ½). In general, *T_c_* is larger, with several hundred degrees compared to the *T_s_* determined visually. This is why our formula permits the prediction of hardness in the high-temperature region as well.

## 4. Conclusions

1. Measurement with commercialized equipment is equivalent with a heat treatment which may influence the validity of high-temperature measurements. Here, we present a relative rapid measurement, reaching the maximum temperature (1100 °C) within half an hour.
2. Due to the high heating rate, all the nonequilibrium alloys (amorphous, over-saturated solid solutions, like HEAs) can be studied in their “as received” state, suppressing or eliminating the time-dependent diffusional effects.
3. A detailed measurement protocol was presented to facilitate the determination and evaluation of high-temperature hardness measurement.
4. The presented device serves as a dilatometer as well, applying the necessary corrections for the dilatation of a quartz tube and tungsten indenter.
5. The presented device is an order of magnitude cheaper than those commercially available.
6. The presented device is easy to build in even a moderately equipped lab.
7. A new formula was presented, permitting researchers to fit the experimental results within the whole temperature range.

We continue to improve the instrument in order to extend the working temperature up to 1200 °C. After a minor modification of the instrument, creep measurements will be performed to test the generally applied mathematical description [16] for the specific cases of refractory high-entropy alloys.

## Figures and Tables

**Table 1:** Data of investigated refractory high-entropy alloys.

	RHEA	a (Angstrom)	VEC	*HV* (kgf/mm^2^)	*Ts* (°C)	*Tc* (°C)	*n*
1	Y25Ti25Zr25Hf25	3.57702	3.75	271.3	610	823	5.53
2	Ti33.33Zr33.33Hf33.34	3.47057	4	310.16	*Ts*1 = 450 *Ts*2 = 890	706	5.8
3	Ti30Zr30Hf30Nb10	3.45071	4.1	336.46	722	803	11
4	Ti25Zr25Hf25Nb25	3.42335	4.24	336.21	860	996	13.1
5	Ti25Zr25Hf25Nb25	3.42335	4.4	415.19	744	830	10.8
6	Ti25Zr25V25Nb25	3.29035	4.5	381.16	520	758	8
7	Ti20Zr20V20Nb20Ta20	3.30111	4.6	398.71	1000	1004	4.91
8	V25Nb25Mo25W25	3.17187	5.5	432.24	1000	1250	12.5

## Data Availability

The original contributions presented in the study are included in the article, further inquiries can be directed to the corresponding author.
